# Exploring the ketogenic diet’s potential in reducing neuroinflammation and modulating immune responses

**DOI:** 10.3389/fimmu.2024.1425816

**Published:** 2024-08-12

**Authors:** Antonietta Monda, Maria Ester La Torre, Antonietta Messina, Girolamo Di Maio, Vincenzo Monda, Fiorenzo Moscatelli, Marida De Stefano, Marco La Marra, Marilena Di Padova, Anna Dipace, Pierpaolo Limone, Maria Casillo, Marcellino Monda, Giovanni Messina, Rita Polito

**Affiliations:** ^1^ Department of Human Sciences and Quality of Life Promotion of the Telematic University “San Raffaele”, Rome, Italy; ^2^ Department of Clinical and Experimental Medicine, University of Foggia, Foggia, Italy; ^3^ Department of Precision Medicine, University of Campania “Luigi Vanvitelli”, Naples, Italy; ^4^ Department of Experimental Medicine, Section of Human Physiology and Unit of Dietetics and Sports Medicine, University of Campania “Luigi Vanvitelli”, Naples, Italy; ^5^ Department of Exercise Sciences and Well-Being, University of Naples “Parthenope”, Naples, Italy; ^6^ Department of Wellbeing, Nutrition and Sport, Pegaso Telematic University, Naples, Italy; ^7^ Department of Humanistic Studies, University of Foggia, Foggia, Italy

**Keywords:** ketogenic diet, neuroinflammation, ketone bodies, central nervous system, immunomodulation

## Abstract

The ketogenic diet (KD) is marked by a substantial decrease in carbohydrate intake and an elevated consumption of fats and proteins, leading to a metabolic state referred to as “ketosis,” where fats become the primary source of energy. Recent research has underscored the potential advantages of the KD in mitigating the risk of various illnesses, including type 2 diabetes, hyperlipidemia, heart disease, and cancer. The macronutrient distribution in the KD typically entails high lipid intake, moderate protein consumption, and low carbohydrate intake. Restricting carbohydrates to below 50 g/day induces a catabolic state, prompting metabolic alterations such as gluconeogenesis and ketogenesis. Ketogenesis diminishes fat and glucose accumulation as energy reserves, stimulating the production of fatty acids. Neurodegenerative diseases, encompassing Alzheimer’s disease, Parkinson’s disease are hallmarked by persistent neuroinflammation. Evolving evidence indicates that immune activation and neuroinflammation play a significant role in the pathogenesis of these diseases. The protective effects of the KD are linked to the generation of ketone bodies (KB), which play a pivotal role in this dietary protocol. Considering these findings, this narrative review seeks to delve into the potential effects of the KD in neuroinflammation by modulating the immune response. Grasping the immunomodulatory effects of the KD on the central nervous system could offer valuable insights into innovative therapeutic approaches for these incapacitating conditions.

## 1 Introduction

**Figure 1:**
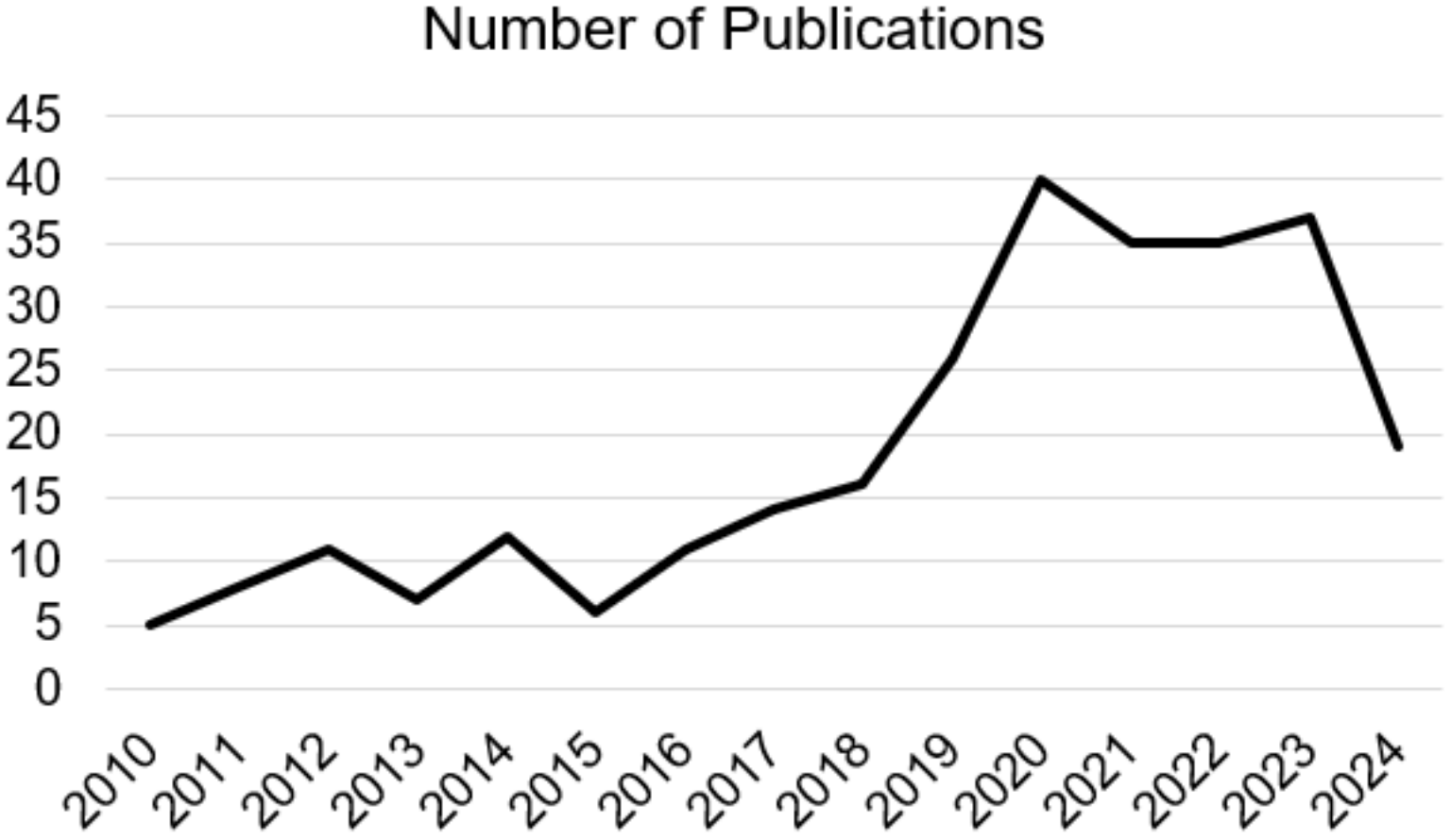
Number of Publications on “ketogenic diet AND neurodegenerative disease” from 2010 to 2024 (in progress).

The ketogenic diet is characterized by a significant reduction in the consumption of carbohydrates and a greater intake of fats and proteins, this type of diet determines a metabolic state called “ketosis”, in which fats are used as the main energy source instead of carbohydrates (1). Many recent studies have identified how the ketogenic diet can induce potential benefits in reducing the risk of certain pathologies such as type 2 diabetes (2), hyperlipidemia (2), heart disease (3) but also cancer (4). Therefore, KD therefore consists of a high intake of lipids, a moderate consumption of a good number of proteins and a low intake of carbohydrates. Generally, KD can thus be divided into the various percentages of macronutrients, i.e. fats which can vary from 60-90% (usually 70-75%) of the entire energy intake, carbohydrates less than 50 g per day (generally represented by 5-10% of total kcal) and proteins with a range from 1.0-1.2 to 1.7 g per kg of body weight (represented by 20% of daily kcal). The main purpose of carbohydrates is to provide energy for various tissues in the body (5). However, when their intake is limited to levels below 50 g/day, subsequent insulin secretion decreases significantly, inducing a catabolic state. Due to this process, glycogen stores are used to the point of exhaustion, triggering a series of metabolic changes, including gluconeogenesis and ketogenesis (6). During ketogenesis, insulin secretion is low due to low blood glucose levels via feedback mechanism, resulting in a reduction in the accumulation of fat and glucose as energy reserves, also triggering further hormonal changes that contribute to an increase of the use of fats as an energy source and subsequent production of fatty acids (7). Neurodegeneration is defined as a pathological condition that mainly affects the brain’s environment at neuronal level. Neurodegenerative diseases are a large group of neurological disorders that affect specific regions of the CNS, having various clinical and pathological characteristics. Conditions in this subgroup include Alzheimer’s disease (AD), Parkinson’s disease (PD), amyotrophic lateral sclerosis (ALS), frontotemporal dementia (FTD), and Huntington’s disease (HD). Although the underlying mechanisms are different, such as the different protein aggregates or genetic variations, they share the hallmark of chronic neuroinflammation (8). Initially, the molecular mechanisms that were recognized as underlying these pathologies were mainly focused on anatomical changes including, as cited previously, protein aggregation such as amyloid beta plaque (Aβ), neurofibrillary tangles (NFTs) and neuronal damage. Subsequently, the presence of possible immune-related proteins in patients with AD was reported for the first time among the causes of neurodegeneration. The causes and pathogenesis are complex and diverse, mainly including protein aggregations, mutations, and infections. Following numerous studies, it has been suggested that the chronic activation of the immune response in the CNS and the presence of high levels of inflammatory factors play a notable role in the emergence and pathogenesis of neurodegenerative diseases (9). Cerebral neuroinflammation is fundamentally characterized by the activation and proliferation of some main immune cells forming part of the CNS such as microglia and astrocytes, subsequently accompanied by the regulation and release of inflammatory mediators (10). Currently, studies on possible therapies are still ongoing and the development of effective therapeutic strategies is essential. Recently, emerging evidence has underlined and highlighted both the potential pathophysiological and clinical benefits of KD in neurodegenerative diseases, indicating it as a possible treatment (11). The protective power of KD is associated with the creation of ketone bodies (KB), fundamental players and factors of this type of diet. In fact, over the years, studies on the neuroprotective and inflammation-modulating effects associated with KD have increased, increasingly deepening research in this field, as well as in other types of pathologies. For about 100 years, KD has been the protagonist of numerous publications in the PubMed search engine, inserting the words “ketogenic diet AND neurodegenerative disease”, with about 259 results from 1924 to 2024 (in progress), noting an increase especially in the last decades starting from the 2010s, with around 245 results (**Figure 1**). In light of these described concepts, and analyzing articles from the last decade, although the complete mechanisms of the influence of KD for the treatment of neurodegenerative diseases remains to be analyzed with further research, the objective of this review was to systematically summarize the information and findings present in scientific literature to support the use of KD as a possible therapeutic approach, in particular for neurodegenerative diseases, or discuss its potential adverse effects.

## 2 Ketogenic diet

**Figure 2:**
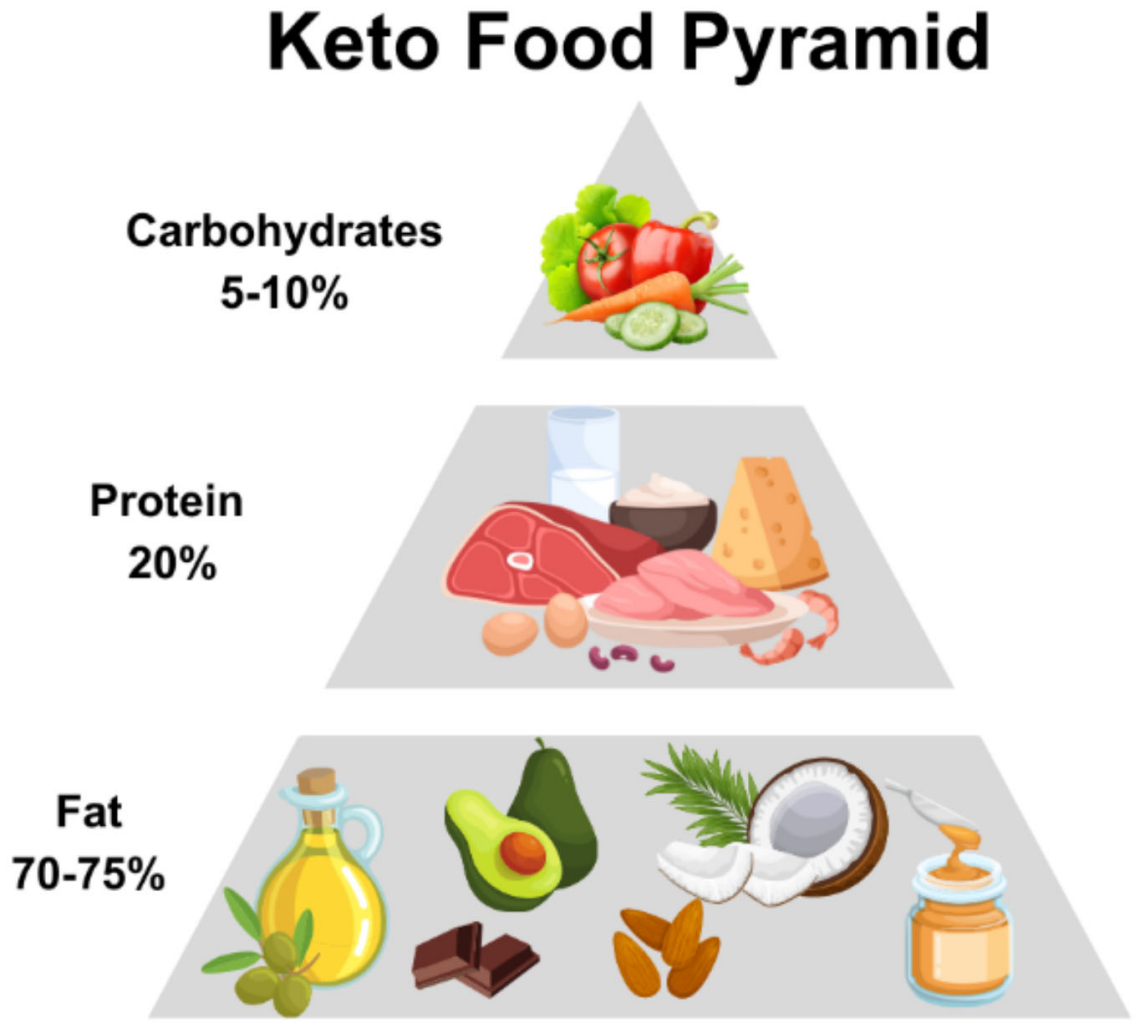
Keto food pyramid.

The ketogenic is a dietary approach which was first used by Russell Wilder as a non-pharmacological treatment for epilepsy in 1921 (12), subsequently coining the term “ketogenic diet”. Through observations, it was noted that this type of dietary regime reduced the frequency and intensity of epileptic seizures in a subgroup of patients characterized by epilepsy who followed this dietary approach (13), in fact for almost a decade, before the use and discovery of specific antiepileptic drugs, the ketogenic diet was considered as one of the best therapeutic options for the treatment of pediatric epilepsy (14). This diet subsequently became popular around 1970s, becoming a potential treatment for various conditions (15), and in weight loss interventions, demonstrating its short-term effectiveness (16). In fact, through the significant reduction in the consumption of carbohydrates and a greater intake of fats and proteins, this type of diet determines a metabolic state called “ketosis”, in which fats are used as the main energy source instead of carbohydrates. Many recent studies have identified how the ketogenic diet can induce potential benefits in reducing the risk of certain pathologies such as type 2 diabetes, hyperlipidemia, heart disease but also cancer (17). Therefore, KD therefore consists of a high intake of lipids, a moderate consumption of a good number of proteins and a low intake of carbohydrates. Generally, KD can thus be divided into the various percentages of macronutrients, i.e. fats which can vary from 60-90% (usually 70-75%) of the entire energy intake, carbohydrates less than 50 g per day (generally represented by 5-10% of total kcal) and proteins with a range from 1.0-1.2 to 1.7 g per kg of body weight (represented by 20% of daily kcal) (18) (**Figure 2**).

Considering a diet characterized by 2000 kcal/day, the total daily intake of carbohydrates would be approximately 20-50 grams per day (18). The main purpose of carbohydrates is to provide energy for various tissues in the body. However, when their intake is limited to levels below 50 g/day, subsequent insulin secretion decreases significantly, inducing a catabolic state. Due to this process, glycogen stores are used to the point of exhaustion, triggering a series of metabolic changes, including gluconeogenesis and ketogenesis (19, 20). During ketogenesis, insulin secretion is low due to low blood glucose levels via feedback mechanism, resulting in a reduction in the accumulation of fat and glucose as energy reserves, also triggering further hormonal changes that contribute to an increase of the use of fats as an energy source and subsequent production of fatty acids (21). Once produced, fatty acids are used to produce ketone bodies (the main metabolites of the ketogenic diet), initially in the form of Acetoacetate (AcAc) and subsequently converted into beta-hydroxybutyrate (BHB) and Acetone, determining the appearance of a metabolic state which is called “nutritional ketosis”, ketotic metabolism that will be maintained until the body is deprived of carbohydrates. Nutritional ketosis can generally be considered “safe” as the production of ketone bodies appears to be in moderate concentrations (approximately 5.0 mmol/l) (22) which have no ability to significantly influence blood pH. Naturally, nutritional ketosis is clearly different from ketoacidosis, which is a serious and life-threatening condition for the individual, with excessively high levels of ketone bodies, resulting in blood acidosis (23). Ketone bodies, once synthesized following the ketosis process, can be used as the main source of energy especially for the main vital organs such as the heart, muscle tissue, kidneys but above all the brain (23, 24). In fact, in this case, ketone bodies have the ability, due to their structure, to cross the blood-brain barrier with the aim of providing alternative energy to the brain, as it is clearly dependent on glucose. Therefore, ketone bodies can play a crucial role in providing energy to the body during periods of low carbohydrate intake such as in the ketogenic diet, allowing the body to maintain energy balance (24). These basic mechanisms, characterizing this type of diet, could be the basis of the treatment of some types of neurodegenerative diseases to exploit the ability of ketone bodies to modulate the complexes implicated in some neurological diseases. Naturally, these mechanisms are still a source of discussion and research in this regard, which will therefore be explored in depth in this review.

## 3 CNS inflammation and neurodegeneration

Neurodegeneration is defined as a pathological condition that mainly affects the brain’s environment at neuronal level. Neurodegenerative diseases are a large group of neurological disorders that affect specific regions of the CNS, having various clinical and pathological characteristics. Conditions in this subgroup include Alzheimer’s disease (AD), Parkinson’s disease (PD), amyotrophic lateral sclerosis (ALS), frontotemporal dementia (FTD), and Huntington’s disease (HD). Although the underlying mechanisms are different, such as the different protein aggregates or genetic variations, they share the hallmark of chronic neuroinflammation (25). Initially, the molecular mechanisms that were recognized as underlying these pathologies were mainly focused on anatomical changes including, as cited previously, protein aggregation such as amyloid beta plaque (Aβ), neurofibrillary tangles (NFTs) and neuronal damage. Subsequently, the presence of possible immune-related proteins in patients with AD was reported for the first time among the causes of neurodegeneration. In recent years, microglial activation was identified as one of the main features of neurodegenerative diseases (26, 27). Therefore, numerous studies and efforts in the field of research have led to a revaluation of the role of inflammation in neurodegeneration, as a triggering and determining cause of the development of neurodegenerative diseases, resulting in immune signalling which may not only be a consequence of protein aggregation in the brain, but also the cause of the accumulation itself in the early stages of the pathological process (28, 29). The immune system, in fact, performs numerous functions in maintaining tissue homeostasis, including for example the elimination of possible pathogens but also the recovery of injuries (30, 31). Generally, the immune response resolves and repairs the tissue injury beneficially and eliminates the infection present. Despite this, in some cases, the ability to reduce and turn off a possible inflammatory stimulus is lacking, therefore, these resolving mechanisms could be insufficient, resulting in chronic inflammation, which leads to the release of neurotoxic factors and an increase in the disease. The causes that determine the birth and duration of chronic inflammation can be multiple, for example depending on endogenous and genetic factors (32, 33), environmental factors (systematic infection, intestinal dysbiosis, senescence processes, nutritional lifestyle) (34, 35), but also a possible genetic susceptibility, for example mutations of apolipoprotein E4 (APOE4), an allele associated with an increased risk of late onset of AD disease (36, 37). Furthermore, recent studies affirm how a group of bioactive lipids, specialized pro-resolving lipid mediators (SPMs), could be induced to promote the resolution and recovery of inflammation. Failure to turn off inflammation would lead to reduced production of SPM, resulting in an increase in chronic inflammatory diseases (38). Furthermore, another fundamental key concept to explore is the microglial role on neurodegenerative disease, in fact, the inflammatory receptors present on the surface of immune cells, in particular glial cells, generally act as sensors to detect possible changes in the homeostasis of the environment. Microglia, macrophages resident in the brain environment, and astrocytes, in fact, are considered as immune cells that are mainly involved in neuroinflammation processes (39). Microglia present proinflammatory or neuroprotective roles in the CNS based on the physiological response following possible external stimuli; Depending on their phenotype, they can be distinguished into the M1 phenotype of the pro-inflammatory type and the M2 phenotype of the anti-inflammatory type. M1 type microglia, activated by nuclear factor κB (NF-κB) but also by STAT3 signal transducers, generate proinflammatory cytokines, and in particular the interleukins IL-1β, IL-6, IL-12, IL-23, cyclooxygenase (COX)-1, COX-2, ROS and nitric oxide (NO) (39). However, in the case of the M2 anti-inflammatory phenotype, when activated they release neurotrophic factors, such as IL-4, IL-10, IL-13 and transforming growth factor-β (TGF-β) (40). Similar to microglia, even astrocytes if activated present a double phenotype (A1 and A2) in order to respond to external pathological agents and therefore contribute to the neuroinflammatory process, resulting in the production of proinflammatory cytokines (such as IL-1β, TNF-α, IL-6 and NO) or anti-inflammatory cytokines (such as IL-4, IL-10, IL-13 and TGF-β) via the STAT3 pathway (40). Microglial activation is also determined by Molecular Patterns associated with DAMP or PAMP Pathogens (41), which lead to the activation of the signal by activating transcription processes and inducing the secretion of inflammatory mediators which in turn amplify further inflammation. Generally, glial cells, if activated, should eliminate possible pathogens by inducing an inflammatory resolution process to eliminate DAMPs or PAMPs, reducing the inflammatory response. However, in some cases, immune cells may not be able to resolve this inflammation which acquires a chronic character, causing neuronal toxicity and favouring protein aggregation. Chronic inflammation leads to increased release of proinflammatory cytokines, including interleukin-6 (IL-6), tumor necrosis factor α (TNF-α), adipokines, and Monocyte chemoattractant protein-1 (MCP -1) (42) which also decrease good mitochondrial function, stimulate the production of ROS (43). Consequently, these inflammatory mediators cause damage at the blood-brain barrier (BBB), entering the brain, inducing persistent chronic neuroinflammation and subsequently causing neurodegeneration (42). For example, in AD, immune cells and therefore microglia, if activated in a state of chronic inflammation, could be involved in the birth of the Aβ plaque by increasing its secretion, inducing the expression of interferon-induced transmembrane protein 3 (IFITM3) which improves the aggregation of β-amyloid plaque (44, 45). This state of inflammation would facilitate a link between the initial aggregation of Aβ and the development of “tau” aggregates with the formation of harmful clusters in the brain that fail to perform their structural stabilizing functions (46, 47). The accumulation of this type of tangles would lead to a further loss of neuronal function in the nerve fiber and, as a final process, to cellular apoptosis. In PD pathology, however, proinflammatory cytokines released by microglial inflammation could induce the phosphorylation of α-synuclein, a key protein in maintaining the production of neurons in the brain, at serine 129 (Ser129) (48), thus promoting degradation of α-synuclein itself (49). Similarly, TDP-43 aggregates, recognized as the main pathogenic factor of ALS, following persistent inflammation, could invade mitochondria, releasing mtDNA and inducing inflammation in neurons through activation of the cGAS/STING pathway (50). Therefore, inflammation at the microglial and neuronal level could have a close relationship with the development of these pathologies.

## 4 The neuroprotective role of the ketogenic diet in inflammation and neurodegeneration: mechanisms and therapeutic potential

Over the years, this diet has been evaluated from various aspects, in an ever more in-depth manner, especially in the field of various pathologies; however, the main primary use, since its discovery, mainly includes neurological diseases having initially been used in epilepsy. The effect of the ketogenic diet on neurological diseases has been widely described in various publications highlighting its effects on neuroprotection, neuroinflammation and neuroplasticity, neurotransmitter function and changes in cellular energy and metabolism among other aspects (51). Neurodegenerative diseases, such as Alzheimer’s disease (AD) and Parkinson’s disease (PD), but also Huntington’s disease (HD) and amyotrophic lateral sclerosis (ALS), are pathologies that are characterized by the reduction and loss progressive and chronic progression of neuronal structure and functions (52, 53). The causes and pathogenesis are complex and diverse, mainly including protein aggregations, mutations, infections. Following numerous studies, it has been suggested that the chronic activation of the immune response in the CNS and the presence of high levels of inflammatory factors play a notable role in the emergence and pathogenesis of neurodegenerative diseases (54). Cerebral neuroinflammation is fundamentally characterized by the activation and proliferation of some fundamental immune cells forming part of the CNS such as microglia and astrocytes, subsequently accompanied by the regulation and release of inflammatory mediators (55, 56). Currently, studies on possible therapies are still ongoing and the development of effective therapeutic strategies is essential. Recently, emerging evidence has underlined and highlighted both the potential pathophysiological and clinical benefits of KD in neurodegenerative diseases, indicating it as a possible treatment option (57). The protective power of KD is associated with the creation of ketone bodies (KB), fundamental players and factors of this type of diet. Following dietary carbohydrate restriction, once KB reach plasma concentrations greater than 4 mmol/L, a shift from the use of glucose as the main brain source of energy to KB occurs (58). KB have a particularity: they can be absorbed into the brain tissue via monocarboxylate transporters (MCT) on microvascular endothelial cells and astrocytes; the transport process by MCTs depends on circulating KB concentrations (57, 59). MCTs are a family of transporters which are linked to protons which passively transport possible metabolic substrates, such as lactate, pyruvate and therefore KB. Four isoforms are the main ones, including MCT1 (SLC16A1), MCT2 (SLC16A7), MCT3 (SLC16A8) and finally MCT4 (SLC16A3), each having affinity for characteristic substrates (60). MCTs are ubiquitously expressed in the brain, as they are the exclusive transporters for KB (38, 41). The main ketone bodies produced in the liver are acetoacetate (AcAc), betahydroxybutyrate (BHB) and acetone (61). Acetoacetate is reduced to BHB by an enzyme “β-hydroxybutyrate dehydrogenase 1” (BHD1); both BHB and AcAc can be transported into the bloodstream or to their respective target organs via MCT transporters (60), while acetone, is produced from a small portion of acetoacetate and then metabolized in the liver as a waste product. Under normal physiological conditions, both BHB and AcAc are excreted at the renal level, while acetone at the pulmonary level (51, 62). MCT1 has a relatively low affinity for BHB, mainly in endotheliocytes and astrocytes of the blood-brain barrier (BBB) (63), while MCT2, with high affinity for BHB, is present almost exclusively at the postsynaptic level of neurons (64) but also with low affinity in astrocytes (65). It is also interesting to underline that according to recent studies FFA can cross the BBB and actively participate in ketogenesis in astrocytes (66). Based on this mechanism, thanks to numerous field research, it has been possible to observe how many neurodegenerative diseases are characterized by disorders of glucose metabolism at the neuronal level. Extremely important it follows that in case of brain trauma, and therefore with the help of ketosis, the number of MCT channels in brain cells increases and allows BHB to be catalyzed and metabolized (67, 68). Furthermore, it has been seen that the application of KD could reduce the demyelination of the myelin sheath of nerve fibers, the death of oligodendrocytes (cells responsible to produce myelin) and the degeneration of axons which could be caused by glucose deficiency (69). Furthermore, ketone bodies and in particular BHB can contribute to the reconstruction of the respiratory chain, as the energy derived from ketone bodies can be assimilated even in the event of damage to the first complex of the respiratory chain (70, 71). In this regard, the caloric deficit typical of KD would show a neuroprotective potential, increasing the number of neuroprotective factors, including brain-derived neurotrophic factor (BDNF) and glial cell line-derived neurotrophic factor (GDNF), neutrophin-3 (NT-3) and molecular chaperones (71). Caloric deficiency would also improve mitochondrial function, reducing the production of reactive oxygen species (ROS) and showing a consequent anti-inflammatory potential given by the inhibition of the activities of cyclooxygenase-2 (COX-2) and inducible nitric oxide synthase (iNOS), as well as by blocking the synthesis of IL-1β, IL-2, IL-4, IL-6, TNFα. and NFκB (72, 73).In this case, the effect of KD was demonstrated in reducing microglial and neuritis activation via inhibition of COX-2 and PPARγ. The condition of ketosis and production of ketone bodies could promote brain autophagy through the activation of sirtuin 1 (SIRT1) and hypoxia-induced factor 1α (HIF-1α) and the inhibition of the mTORC1 complex, preventing neurodegenerative disorders by eliminating protein aggregates or damaged mitochondria (74, 75). For example, studies have shown that KD could reduce the number of beta-amyloid plaques and other metabolic byproducts in brain tissue (76). The KD and the ketone bodies derived from it would finally determine the restoration of the integrity of the BBB following the increase in connexin-43 (Cx43) useful in the construction of the BBB and the MCT and GLUT transporters (glucose transporters) (77, 78). KD could determine the polarization of microglia, upon activation, from M1 to M2 by modifying the inflammatory environment by reducing the production of proinflammatory cytokines, including TNF-α, IL-1β and IL-6 and molecular patterns such as TLR4 and NF-κB, and upregulating the production of anti-inflammatory cytokines such as TGF-β (79). The result is therefore a broad and favourable spectrum of KD effects (**Table 1**), although the mechanisms still need to be verified and better researched.

**Table 1:** The molecular mechanisms involved in the modulation of inflammation and neurodegeneration by KD.4.1 possible neuroprotective effect of ketone bodies.

Biological activity	Reference
Energy metabolism mediated by by ketone bodies in cases of glucose metabolism disorders.	(68, 69)
KD could reduce the demyelination of the myelin sheath of nerve fibers, death of oligodendrocytes and degeneration of axons.	(69)
KD and BHB contribute to the reconstruction of the respiratory chain even in the event of damage to the first complex	(70, 71)
KD increase the number of neuroprotective factors, as BDNF, GDNF, NT-3 and molecular chaperones.	(71)
KD and Caloric deficiency improve mitochondrial function, reducing the production of ROS.	(72)
KD reduce iNOS, blocking the synthesis of IL-1β, IL-2, IL-4, IL-6, TNFα and NFκB.	(72, 73)
KD and ketone bodies promote brain autophagy through activation of SIRT1 and HIF-1α, and inhibition of the mTORC1 complex, eliminating protein aggregates or mitochondria damage.	(74, 75)
KD and the ketone bodies determine the restoration of the integrity of the BBB with the increase in Cx43.	(77, 78)
KD determine the polarization of microglia from M1 to M2 reducing the production of TNF-α, IL-1β, IL-6 and TLR4 and NF-κB, and upregulating the production of TGF-β.	(79)

**Figure 3:**
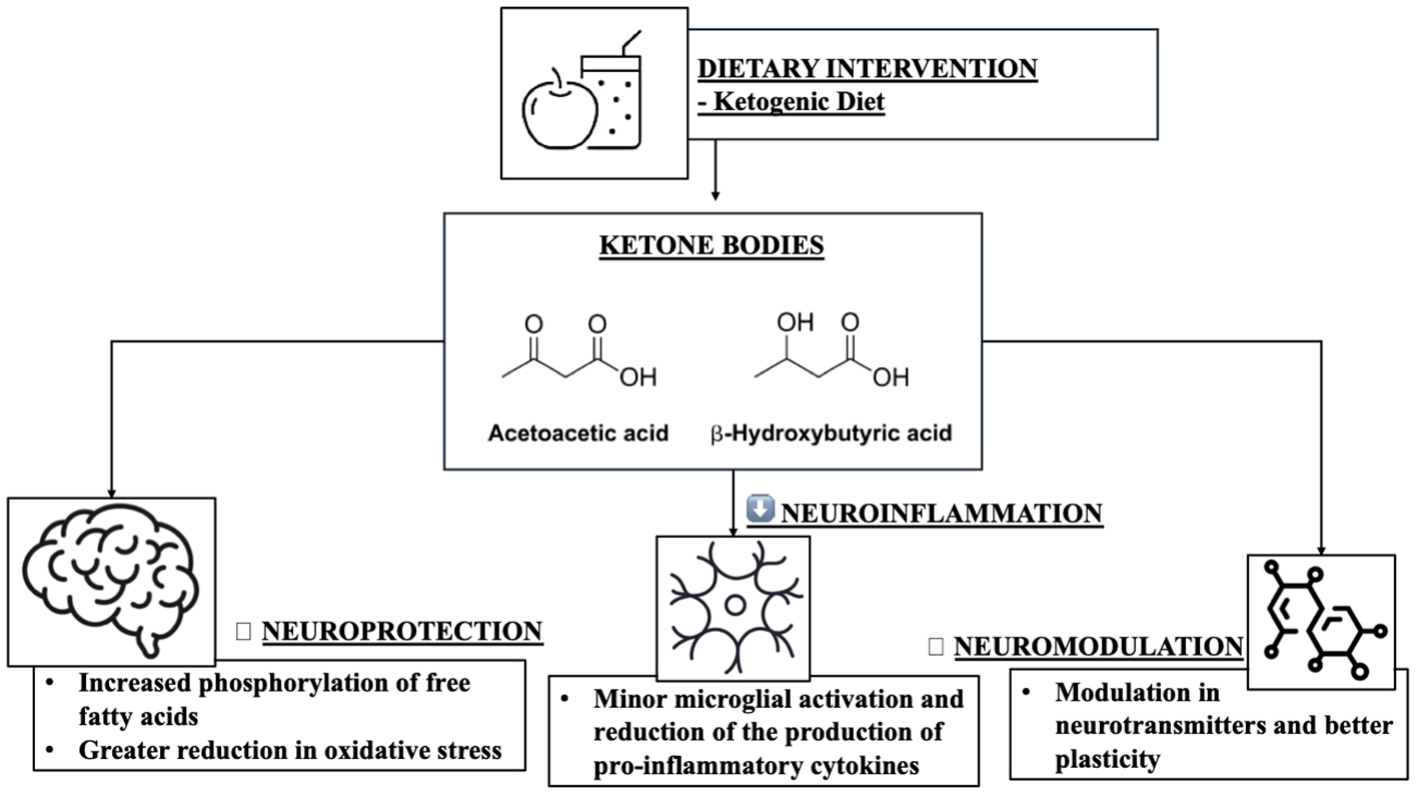
The role of ketogenic diet in neuroinflammation and neuromodulation.

Once absorbed into the brain, ketone bodies can play various neuroprotective roles in the CNS. Mainly, since KB are produced following a carbohydrate and dietary restriction with consequent inhibition of glycolysis, this would improve insulin sensitivity and therefore glucose tolerance, often related to the onset of age-related conditions (80, 81). Secondly, KB could compensate for possible mitochondrial dysfunctions in neurons and especially in glial cells by improving mitochondrial respiratory functions (according to a recent study BHB regulates “nicotinamide adenine dinucleotide” by decreasing the NAD+/NADH ratio) and increasing ATP production (82). Furthermore, increased mitochondrial function could result in blocking complex I (CI) in the etiological mechanism of PD (83), and similarly alleviating the production of reactive oxygen species (ROS) resulting in reduced inflammatory responses through various pathways. KB production may also be able to prevent neuronal apoptosis via the sirtuin (SIRT)-1 signalling pathway, i.e. KB may reduce SIRT-1-mediated hyperacetylation of the transcription factor p53 and consequently down -regulating proapoptotic protein (BAX) and up-regulating anti-apoptotic proteins (Bcl-2 and Bcl-xL) in animal models (84). Furthermore, KB are particularly involved in neuroinflammation processes at the glial level (85). Resident glial cells of the central nervous system (microglia and astrocytes) mediate neuroinflammation processes by regulating the production of inflammatory cytokines, ROS, and chemokines (86). Based on the progress of the inflammatory response, neuroinflammation is divided into acute and chronic. Generally, an effective inflammatory response allows the elimination of invading pathogens, promoting the repair of damaged tissues (87). However, an uncontrolled neuroinflammation process could induce a chronic inflammatory response, characterized by hyperactivation of microglia and the uncontrolled production of neurotoxic factors but also by neuronal and glial cell destruction, which together would accelerate the deterioration of the initial condition (88). Microglia and therefore, are immune cells mainly involved in neuroinflammation (89, 90), considered as resident macrophages in the brain, play a fundamental role in neuroinflammation (91). Microglia exhibit pro-inflammatory or neuroprotective roles in the CNS based on their phenotype (proinflammatory M1 phenotype and anti-inflammatory M2 phenotype) (92). M1-type microglia (classical-type activation) are activated via pro-inflammatory nuclear factor κB (NF-κB) and signal transducers of transcription 3 (STAT3) signalling pathways to generate proinflammatory cytokines, such as example interleukin (IL)-1β, IL-6, IL-12, IL-23, cyclooxygenase (COX)-1, COX-2, ROS and nitric oxide (NO). While M2 type microglia (alternative type activation), are activated with the aim of releasing neurotrophic factors, such as IL-4, IL-10, IL-13 but also transforming growth factor-β (TGF-β) (93). In contrast, astrocytes, also part of glial cells, can regulate the brain environment, maintaining neurotransmitter homeostasis, controlling synapse formation, and forming distinctive perivascular channels to eliminate potentially neurotoxic agents. Being very similar to microglia, they too can be activated into A1 phenotype and A2 phenotype to respond to pathological insults and participate in the neuroinflammatory process (94). Over time, it has been stated that the neuroinflammatory process certainly contributes to the birth and pathological progression of various neurodegenerative diseases (95), as being neuroimmune cells, microglia and astrocytes modulate the inflammatory response, releasing proinflammatory cytokines or anti-inflammatory (95). BHB, in this context, promotes microglial branching typical of M2 alternative type activation, with anti-inflammatory properties by acting on protein kinase B (Akt)-small RhoGTPase (96). Currently, there is growing evidence suggesting that KD and KB play neuroprotective roles by regulating central and peripheral inflammatory mechanisms (60). In addition to being substrates for glial protective purposes, KBs can also be active as mediators of intracellular signalling, as they participate in intracellular signalling cascades by regulating neuroinflammation directly or indirectly (97). Hydrocarboxylic acid receptor 2 (HCA2), which can also be called G-coupled protein receptor (GPR) 109A, is a receptor abundantly present in microglia, macrophages but also in dendritic cells. Its activation determines a greater action by COX-1 which in turn generates prostaglandin D2 (PGD2), a factor alleviating neuroinflammation (98). BHB being a specific ligand of HCA2, may be able to activate it starting from a concentration of approximately 0.7 mmol/L, like human nutritional ketosis (99, 100). Furthermore, BHB, by binding to HCA2, also inhibits the production of proinflammatory cytokines and enzymes via the NF-κB pathway in activated microglia pretreated with BHB and lipopolysaccharide (LPS) (101). The activation of HCA2 by BHB would also facilitate the reduction of endoplasmic reticulum stress, the activity of the NLRP3 inflammasome and finally the levels of IL-1β and IL-18 (102). Furthermore, KB can therefore alter the balance of the release of inhibitory and excitatory neurotransmitters, crucial for long-term neuronal survival; it has in fact been demonstrated that AcAc inhibits the uptake of glutamate into presynaptic vesicles, decreasing excitotoxicity (103). Similarly, BHB attenuates the process of autophagy resulting in degradation enhancing neuronal damage (104). It is interesting to consider that 3-Hydroxy-3-Methylglutaryl-CoA Synthase 2 (HMGCS2), i.e. the control enzyme in ketogenesis, would induce the autophagic clearance of the β-amyloid precursor protein, which is mediated by the metabolism of AcAc or and by the mTOR signalling pathway, suggesting that HMGCS2 and therefore AcAc inhibits the possible production and deposition of amyloid β (Aβ) (105). Finally, as initially mentioned, KD and therefore KB can improve insulin sensitivity and the glycemic profile (106, 107), in this case BHB and AcAc slow down insulin glycation, the accumulation of Advanced glycation end products (AGEs) (108, 109) and liposomal lipid peroxidation, thus preventing microglial apoptosis (110). Therefore, it can be hypothesized that the anti-inflammatory effects of KB could reduce the development of neurodegenerative diseases in multiple aspects (**Table 2** and **Figure 3**).

**Table 2:** Role of ketone bodies involved in neuroinflammation and neurodegeneration.

Biological Activity of KB	Reference
KB compensate mitochondrial respiratory dysfunctions (neurons and glial cells), BHB decrease NAD+/NADH ratio increasing ATP production.	(96)
KB increase mitochondrial function, blocking complex I (CI) involved in etiological mechanism of PD.	(97)
KB is able to prevent neuronal apoptosis via SIRT-1 signalling pathway.	(98)
KB is able to prevent neuronal apoptosis via SIRT-1 signalling pathway, reduce p53 and BAX, up-regulating Bcl-2 and Bcl-xL.	(98)
KB are active as mediators of intracellular signaling, BHB binds to HCA2 regulating homeostasis, inhibiting the production of proinflammatory cytokines and enzymes via NF-κB in microglia.	(101)
Binding of BHB to HCA2 facilitates reduction of endoplasmic reticulum stress, NLRP3 inflammasome activity, and IL-1β and IL-18 levels.	(102)
AcAc inhibits glutamate uptake into presynaptic vesicles, decreasing their excitotoxicity.	(103)
KB improve insulin sensitivity and the glycemic profile, BHB and AcAc slow down insulin glycation.	(104, 105)
KB decrease the accumulation of advanced glycation end products (AGEs).	(106, 107)
KB decreases liposomal lipid peroxidation, preventing microglial apoptosis.	(108–110)

## 5 Discussion

Neuroinflammation and immune activation represent crucial components in the pathogenesis of various neurological disorders, including neurodegenerative diseases. The emerging role of the ketogenic diet (KD) in modulating these processes has sparked considerable interest and debate within the scientific community. One aspect of neuroinflammation involves the activation of microglia, the resident immune cells of the central nervous system (CNS). Microglia play a dual role in neuroinflammation, exhibiting both pro-inflammatory (M1 phenotype) and anti-inflammatory (M2 phenotype) responses. Chronic activation of pro-inflammatory microglia contributes to the release of neurotoxic factors, exacerbating neuronal damage and accelerating disease progression. On the other hand, the activation of anti-inflammatory microglia promotes tissue repair and neuroprotection. Recent studies have suggested that the ketogenic diet may exert profound effects on microglial activation and polarization. Ketone bodies, particularly beta-hydroxybutyrate (BHB), have been shown to modulate microglial phenotype (66), shifting them towards the anti-inflammatory M2 phenotype. This polarization may be mediated through various mechanisms, including the inhibition of NF-κB signaling and the activation of hydroxycarboxylic acid receptor 2 (HCA2) (99, 100). By promoting the M2 phenotype, ketone bodies may attenuate neuroinflammation and mitigate neuronal damage in neurodegenerative diseases. Furthermore, the ketogenic diet has been implicated in reducing the production of pro-inflammatory cytokines and chemokines, such as interleukin-1β (IL-1β) and tumor necrosis factor α (TNF-α) (102). These inflammatory mediators play pivotal roles in propagating neuroinflammation and exacerbating neuronal injury. By dampening the release of these pro-inflammatory factors, the ketogenic diet may exert anti-inflammatory effects within the CNS, thereby mitigating the pathological processes associated with neurodegenerative diseases. Moreover, ketone bodies have been shown to modulate other aspects of the immune response, including the regulation of astrocyte function and the promotion of resolution of inflammation. Astrocytes, like microglia, play crucial roles in maintaining CNS homeostasis and responding to pathological insults. The ketogenic diet may influence astrocyte activation and polarization, thereby exerting additional effects on neuroinflammation and immune activation (30, 31). While accumulating evidence suggests a potential role for the ketogenic diet in modulating neuroinflammation and immune activation, several questions and challenges remain. For instance, the optimal composition and duration of the ketogenic diet required to elicit therapeutic effects in neurodegenerative diseases are yet to be fully elucidated. Moreover, the precise mechanisms underlying the immunomodulatory effects of ketone bodies within the CNS warrant further investigation. Overall, the immunomodulatory effects of the ketogenic diet (KD) represent a fascinating area of research with potential implications for various health conditions, including neurological disorders, metabolic diseases, and inflammatory conditions. One of the primary mechanisms by which the KD exerts its immunomodulatory effects is through the inhibition of inflammatory pathways. Ketone bodies, particularly beta-hydroxybutyrate (BHB), have been shown to inhibit the activation of the NLRP3 inflammasome (103), a key mediator of inflammation. By dampening NLRP3 activation, BHB reduces the production of pro-inflammatory cytokines such as interleukin-1β (IL-1β) and interleukin-18 (IL-18) (102), thereby attenuating the inflammatory response. The KD has been shown to modulate the function of various immune cells, including macrophages, T cells, and dendritic cells. Ketone bodies can influence the polarization of macrophages towards an anti-inflammatory M2 phenotype, which is associated with tissue repair and resolution of inflammation. Additionally, the KD may promote the expansion of regulatory T cells (Tregs), which play a critical role in maintaining immune tolerance and suppressing excessive inflammation. Oxidative stress is closely linked to inflammation, and the KD has been shown to reduce oxidative stress through multiple mechanisms. Ketone bodies possess antioxidant properties and can scavenge reactive oxygen species (ROS) (86), thereby mitigating oxidative damage to cells and tissues. By reducing oxidative stress, the KD may help attenuate inflammation and prevent tissue injury. Furthermore, emerging evidence suggests that the KD can modulate the composition and function of the gut microbiota, which plays a crucial role in regulating immune function. By promoting the growth of beneficial bacteria and suppressing the growth of pathogenic microbes, the KD may exert indirect immunomodulatory effects through its impact on the gut microbiota. Metabolic dysregulation is intricately linked to inflammation, and the metabolic changes induced by the KD may contribute to its immunomodulatory effects. By promoting ketogenesis and shifting cellular metabolism away from glycolysis, the KD alters cellular signaling pathways involved in inflammation and immune activation. In addition to its systemic immunomodulatory effects, the KD has been shown to exert specific effects on neuroinflammation within the central nervous system (CNS). Ketone bodies can penetrate the blood-brain barrier and modulate microglial activation, thereby reducing neuroinflammation and providing neuroprotection in various neurological disorders. Overall, the immunomodulatory effects of the ketogenic diet are multifaceted and involve complex interactions between metabolic, inflammatory, and immune pathways. In conclusion, the ketogenic diet holds promise as a potential therapeutic strategy for mitigating neuroinflammation and immune activation in neurodegenerative diseases. By targeting microglial and astrocyte function, as well as modulating inflammatory signaling pathways, the ketogenic diet may offer novel avenues for the treatment of these devastating disorders. However, further research is needed to fully understand the complex interplay between the ketogenic diet and neuroinflammation, paving the way for the development of effective therapeutic interventions (111).
